# Hierarchical Composites to Reduce *N*-Nitrosamines in Cigarette Smoke

**DOI:** 10.3390/ma8031325

**Published:** 2015-03-20

**Authors:** Yan Yan Li, Yi Cao, Ming Bo Yue, Jing Yang, Jian Hua Zhu

**Affiliations:** Key Laboratory of Mesoscopic Chemistry of MOE, College of Chemistry and Chemical Engineering, Nanjing University, Nanjing 210093, China; E-Mails: dg1324052@smail.nju.edu.cn (Y.Y.L.); Caoyi_nju@163.com (Y.C.); mingboyue@126.com (M.B.Y.); yangjingsz01@163.com (J.Y.)

**Keywords:** zeolite, hierarchical composite, cigarette smoke, *N*-nitrosamine, environment protection

## Abstract

In order to reduce the harmful constituents in cigarette smoke, two hierarchical composites were synthesized. Based on, zeolites HZSM-5 or NaY fragments were introduced into the synthetic system of mesoporous silica SBA-15 or MCM-41 and assembled with the mesoporous materials. These porous composites combine the advantages of micro- and mesoporous materials, and exhibit higher effects than activated carbon on reducing tobacco specific nitrosamines (TSNA) and some vapor phase compounds in smoke.

## 1. Introduction

Tobacco and tobacco products are widely consumed throughout the world, and the pollution and health hazards caused by smoking are a serious problem [1]. Cigarette smoke contains more than 5200 components [2], among them 69 carcinogens have been identified and several are tumor promoters or co-carcinogens. Therefore, it is imperative to reduce the harmful constituents in cigarette smoke to protect the health of humans. As a common consensus, cigarette smoke is composed of a vapor phase and a particulate phase [3]. The vapor phase can be considered as a mixture of a smoke aerosol. The particulate phase is composed of numerous semi- and non-volatile compounds, with particle sizes ranging from 0.1 to 1.0 μm in diameter [4]. Normally, the carcinogenic and toxic constituents such as carbon monoxide and volatile *N*-nitrosamines *etc.* are hidden in the vapor phase of the mainstream smoke inhaled by the smoker [3], while nicotine, tobacco specified *N*-nitrosamines (TSNA), and polynuclear aromatic hydrocarbons (PAHs) *etc.* are part constituents of the particulate matter. Besides, some components like phenol can be found in both vapor phase and particulate phase, and some semi-volatile *N*-nitrosamines and volatile compounds can also be detected in the particulate phase as an aerosol type [3]. Consequently, the existence status of these carcinogenic and toxic components in cigarette smoke is very complicated. 

In order to protect humans from the health risk caused by smoking, it is necessary to reduce the concentration of the harmful constituents in cigarette smoke. Although some carcinogens such as TSNA are formed during the curing and aging process of tobacco [3,5], most of these compounds are generated by pyrosynthesis in the combustion of the cigarette [6]. Apart from utilization of gamma irradiation [7], porphyrins [8], piperazine or monoethanolamine [9], nanostructural titanates [10], and bacterial reduction [11], there are two main strategies for using additives to reduce the carcinogenic components in the mainstream smoke. One-way is to mix the additives, such as zeolite NaY [12,13,14,15,16,17,18], with tobacco fibers directly which are activated when the hot zone in the burning cigarette approaches them. Though these additives are always innoxious and stable at high temperature, they are unavoidably involved in the combustion of cigarettes, which brings some uncertain factors to the burning routine. The other method is to put the additives into the cigarette filter tips in order to strengthen the adsorption performance. Activated carbon is a common filter additive though it cannot selectively adsorb carcinogenic components such as nitrosamines [2,19,20]. Zeolite is another kind of additive with selective adsorptive ability to reduce the carcinogens in smoke [13]. Especially, zeolite NaY was found to trap 85% of *N*-nitrosopyrrolidine (NPYR) at 453 K when NPYR passed through it [21]. Due to the limitation of micropore size, however, zeolites fail to trap bulky nitrosamines such as TSNA. On the contrary, mesoporous silica material, such as SBA-15, has a higher activity than zeolite NaY for degrading bulky TSNA such as *N*-nitrosonornicotine (NNN), but is less active for adsorbing volatile nitrosamines with a smaller molecular size because these mesoporous materials lack a fine geometric confinement [14]. In order to overcome the inherent weakness of micro- and mesoporous materials and combine their advantages, the concept of synthesizing a composite with a hierarchical porous structure to reduce the harm of smoking has naturally been considered, and many attempts have been carried out on this subject [22,23,24]. 

Unlike the assembly of hierarchical cracking catalysts where the mesopores are in the outer side and the micropores in the inner side [25,26,27], in the present study zeolites fragments were introduced into the synthetic system of mesoporous silica materials with reference to our previous investigations [14,22,23,24]. After surviving from solution, these zeolite fragments are combined with the framework of the mesoporous material. In order to evaluate the performance of these porous composites for adsorption, three volatile *N*-nitrosamines with different molecular diameter were selected for gaseous adsorption [28]. Additionally these hierarchical composites were also used as filter additives to reduce the carcinogenic constituents in mainstream smoke.

## 2. Results and Discussion

### 2.1. The Structure Properties of Composite Porous Materials

**Figure 1:**
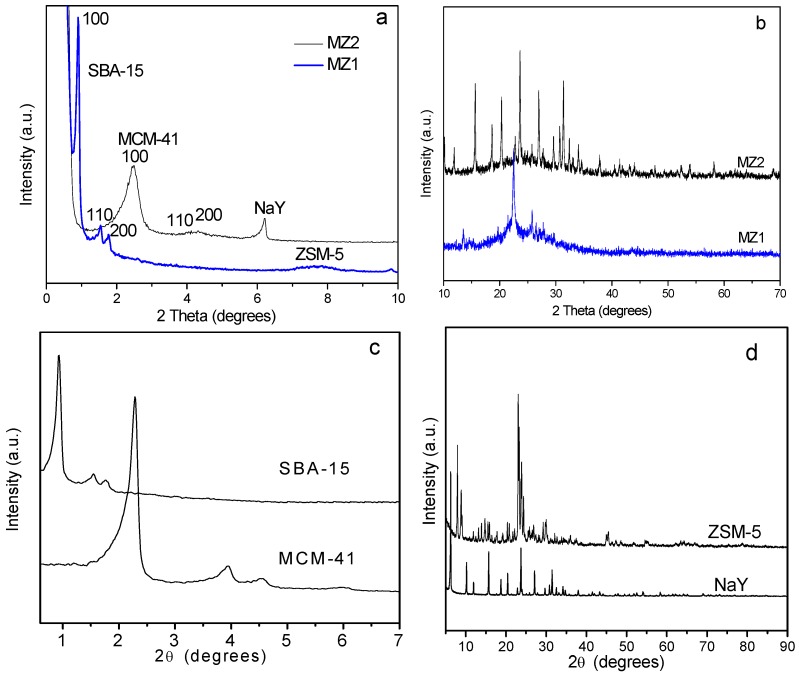
(**a**,**c**) Low-angle and (**b**,**d**) wide-angle XRD of the synthesized samples.

The XRD profiles of zeolite NaY, HZSM-5 and mesoporous silica MCM-41, SBA-15 as well as hierarchical composites MZ1 and MZ2 are provided in Figure 1. In the low-angle region (Figure 1a), the MZ1 sample exhibited three well-resolved diffraction peaks indexed to the (100), (110) and (200) reflections that are characteristic XRD patterns of 2D-hexagonal pore ordering in the *P*6*mm* space group [29], closely matching the XRD pattern of SBA-15. There were some residual diffraction peaks of ZSM-5 zeolite in the high-angle XRD patterns of MZ1 (Figure 1b), reflecting the survival of zeolite fragments from the strong acidic synthetic system along with the existence of a microporous structure in these fragments; the amorphous swell peak was attributed to mesoporous silica [22,23]. Owing to the insertion or blockage of HZSM-5 zeolite fragments in the channel of SBA-15, the XRD diffraction intensity of MZ1 sample was slightly reduced, but this sample still kept the similar mesoporous structure of SBA-15. 

**Figure 2:**
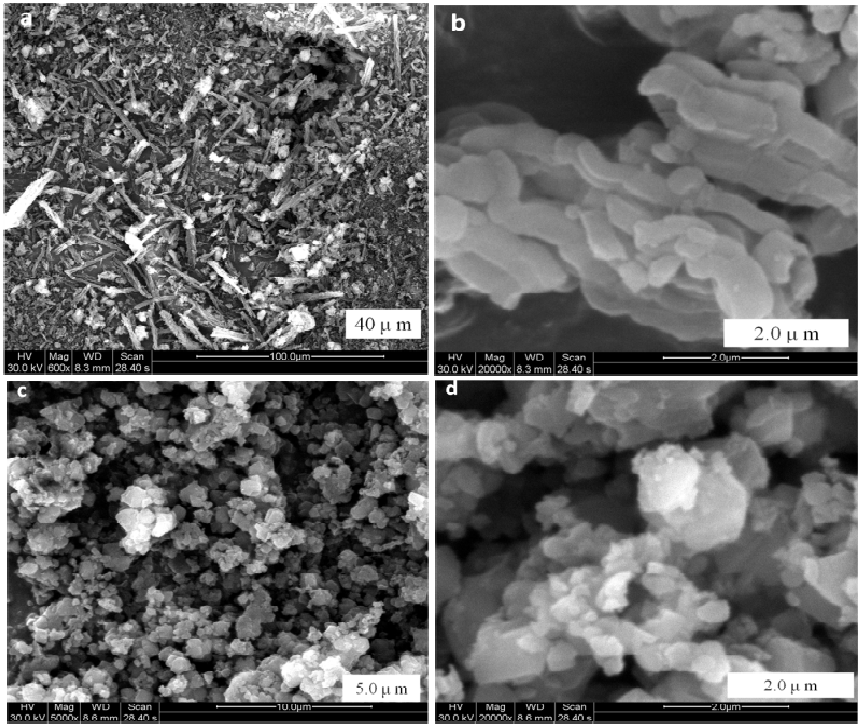
Scanning electron micrographs (SEM) images of MZ1 (**a**,**b**) and MZ2 (**c**,**d**) samples.

Figure 1b presents the XRD experimental result of the MZ2 sample. Compared with the XRD profile of MCM-41, the XRD patterns of MZ2 sample also showed special (100), (110) and (200) diffraction peaks. Introduction of zeolite NaY into the synthetic system enabled aluminum to be incorporated into the lattice of MCM-41, and the intensity of the 100 plane was lower. Meanwhile, the 110 and 200 peaks were overlapped into a broad hump. These two phenomena show that the introduction of zeolite NaY brings slight deterioration to MCM-41, which decreases its ordering and structural uniformity to some degree. Nonetheless, MZ2 still maintained the mesoporous structure similar to that of MCM-41. The high-angle XRD patterns show the clear XRD diffraction peaks of zeolite NaY on the MZ2 sample, which confirms the survival of zeolite NaY fragments in the alkali synthetic system. As a consequence, the MZ2 sample possessed both a mesoporous and a microporous structure. Revealed by the scanning electron micrographs (SEM) of MZ1 and MZ2 samples (Figure 2), it is very clear that MZ1 and MZ2 have different morphology. The former has a fiber-like morphology in microcosm and its particle size is about 2–4 μm, whereas the latter consists of numerous particles deposited together with a small size in the range of 0.5–2 μm. Moreover, there were lots of accumulated pores among the aggregations of MZ2 and their sizes were around 10 μm (Figure 2c,d).

**Figure 3:**
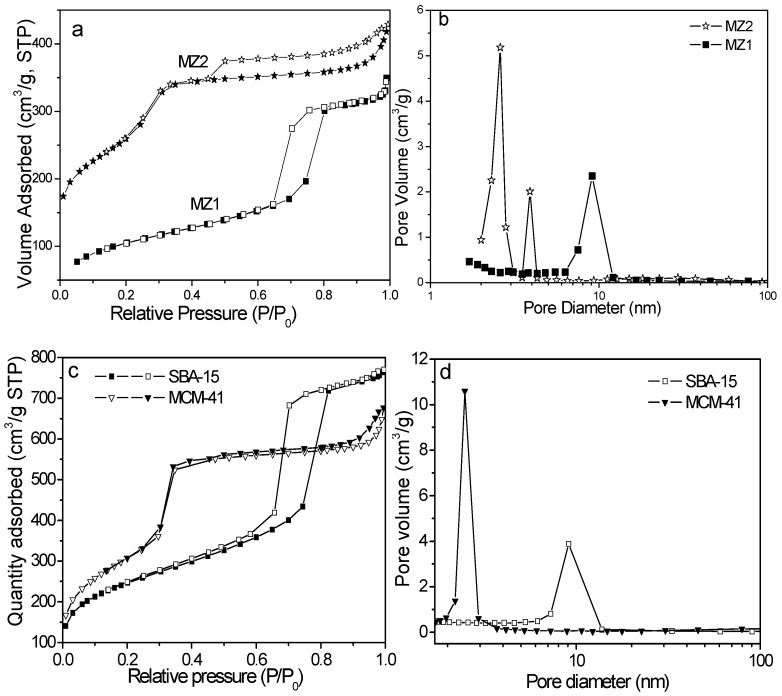
(**a**,**c**) Adsorption-desorption isotherms of nitrogen at 77 K for MZ1, MZ2, SBA-15 and MCM-41 samples; (**b**,**d**) Pore-size distribution of these materials.

Figure 3a represents the N_2_ adsorption-desorption isotherm of MZ1 sample. It is well known that SBA-15 exhibits an isotherm with an H1 type hysteresis loop at high relative pressure [29], nonetheless the MZ1 sample possessed the H2 type hysteresis loop, mirroring the pore blocking effects present in it, because the MZ1 sample comprised an open and closed cylindrical mesoporous containing some plugs [30]. Together with the high-angle XRD patterns of MZ1 as mentioned above, it is safe to infer that ZSM-5 fragments have become inserted and/or blocked the cylindrical mesoporous channels to reduce the pore sizes, and moreover, these incomplete fragments may retain some of the microstructure of parent zeolites as characterized by XRD patterns (Figure 1b). On the other hand, the fragments seem to be well dispersed in the mesostructure of the MZ1 sample because only a slight hindrance of pore was detected: the MZ1 sample had a BET surface area of 545 m^2^ g^−1^, referring to the nitrogen adsorption, while the average pore diameter was about 9.1 nm (Table 1), a little smaller than that of SBA-15 (9.3 nm, Figure 3d). Therefore, the Al-containing SBA-15 composite with a mesoscopic order and some of the microstructure of the ZSM-5 zeolite was obtained as expected.

**Table 1:** Physical properties of porous materials.

Sample	S_BET_ *^a^* (m^2^ g^−1^)	V_t_ *^b^* (cm^3^ g^−1^)	V_mic_ *^c^* (cm^3^ g^−1^)	V_meso_ *^d^* (cm^3^ g^−1^)	D_BJH_ *^e^* (nm)
MZ1	545	0.62	0.09	0.53	9.10
MZ2	916	0.61	0.05	0.56	2.68

Notes: *^a^* S_BET_ = BET surface area; *^b^* V_t_ = total pore volume; *^c^* V_mic_ = micropore volume; *^d^* V_meso_ = mesopore volume; *^e^* D_BJH_ = average pore diameter by BJH calculation.

Figure 3a also displays the N_2_ adsorb-desorption isotherm of MZ2 sample. This curve was a type IV isotherm with one step at P/P_0_ of about 0.34, which is the well-known capillary condensation of the MCM-41-like mesoporous material. Zeolite NaY fragments survived from the alkali synthetic solution and kept their micropores in the MZ2 composite, slightly decreasing the surface area and porosity. Also, the existence of a significant hysteresis loop in the isotherm indicates that zeolite NaY fragments partly blocked the mesopore system.

Table 1 lists the structure parameters of two composites, and Figure 3b shows their pore size distribution curves. The MZ2 sample possessed a larger specified surface area (916 m^2^ g^−1^) but a smaller pore size (2.6 nm) than MZ1. Incorporation of HZSM-5 or NaY zeolite fragments enabled MZ1 or MZ2 samples to have a considerable micropore volume, which is helpful for the adsorption of volatile nitrosamines as discussed later.

**Figure 4:**
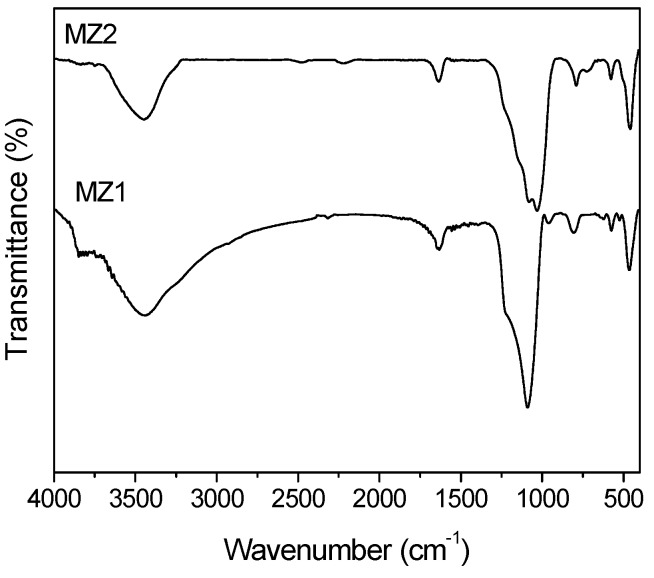
Infrared spectra of MZ1 and MZ2 samples.

Figure 4 illustrates the IR spectra of MZ1 and MZ2 samples in the mid-IR region. The MZ1 sample showed an (Si-O-Si) asymmetrical vibration band at 1090 cm^−1^ and a symmetrical (Si-O-Si) vibration band at 806 cm^−1^. Similarly, the MZ2 sample exhibited these two vibration bands at 1031 cm^−1^ and 791 cm^−1^, respectively. The band at 960 cm^−1^ of the MZ1 sample could be assigned to the asymmetrical (Si-O) stretching mode of ≡Si-O-H^+^ group [31,32], but the MZ2 sample did not show this band. This difference is attributed to the synthetic condition: the MZ1 sample was synthesized in an acid system so the H^+^ proton is beneficial for forming the ≡Si-O-H^+^ group, while the MZ2 sample was obtained from an alkaline environment, which provides the OH^−^ group for the reaction. Thus, it is normal for MZ2 to lack the IR signal of the ≡Si-O-H^+^ group at about 960 cm^−1^. In addition, both of the MZ1 and MZ2 samples exhibited a band at around 575 cm^−1^, originating probably from the vibration of octahedral Al-O [33]. SBA-15 and MCM-41 are mesoporous silica so they do not have the IR signals of Al-O, but MZ1 or MZ2 did have the Al-O signal because their synthesis used HZSM-5 or NaY zeolite fragments as the raw material. Aluminum could be introduced into these silica materials in this way while some zeolite fragments survived in the composite. Therefore, the IR signal of the Al-O band originated from the Al-O structure of the zeolite fragments and/or the Al-containing mesoporous silica.

### 2.2. Adsorption of Volatile N-Nitrosamines from the Gaseous Phase

Table 2 demonstrates the instantaneous adsorption of three volatile *N*-nitrosamines on MZ1 and MZ2 samples at 338 K. In this experiment, we set three distinguishing features to reflect the adsorptive ability of samples used as the filter additives: firstly, the sample was not thermally activated; secondly, the adsorption was performed at 338 K that is close to the temperature of the filter when the cigarette is puffed; thirdly, the contact time between the nitrosamine and the sample was very short, less than 0.1 s [28]. Under these harsh conditions, MZ1 and MZ2 samples showed excellent adsorptive abilities to NPYR and NHMI, and both composites trapped all of the NPYR or NHMI in the gas stream (Table 2), as the zeolite NaY but better than ZSM-5 which adsorbed only about half of the nitrosamines [28], but dramatically superior to mesoporous silica such as SBA-15 which only captured 10%–37% of volatile nitrosamines in the gas stream [34]. The adsorptive ability of MZ1 and MZ2 samples for NDMA was about 98%, slightly inferior to zeolite NaY (100%) but much superior to NaZSM-5 (62%, [28]), let alone the SBA-15 (about 20%, [34]). NDMA is the smallest nitrosamine with a molecular diameter of 0.42 × 0.36 nm^2^, smaller than that of NPYR (0.42 × 0.54 nm^2^) and NHMI (056 × 0.53 nm^2^) [28]. Therefore, the pore size of mesoporous silica such as SBA-15 and MCM-41 is too large to confine the volatile nitrosamines effectively [22,24]. MZ1 or MZ2 samples contained zeolite fragments whose pore size is relatively small and fit for adsorbing these tiny nitrosamines so that the hierarchical composites could efficiently trap these small molecules in the gas flow because they combine the large capacity of mesoporous materials with the effective confinement of zeolites. Although the adsorption of NDMA by these two hierarchical composites was about 98%, it will not affect their function as filter additives because there is only a trace amount of volatile nitrosamines in the mainstream smoke [3].

**Table 2:** Adsorption of three volatile *N*-nitrosamines by MZ1 and MZ2 samples.

Sample	NDMA		NPYR		NHMI	
	Passed (mmol g^−1^)	Adsorbed (mmol g^−1^)	Passed (mmol g^−1^)	Adsorbed (mmol g^−1^)	Passed (mmol g^−1^)	Adsorbed (mmol g^−1^)
MZ1	1.67	1.64	1.13	1.13	0.74	0.74
MZ2	1.63	1.61	1.13	1.13	0.74	0.74

Notes: NDMA: *N*-nitrosodimethylamine; NPYR: *N*-nitrosopyrrolidine; NHMI: *N*-nitrosohexamethyleneimine.

**Figure 5:**
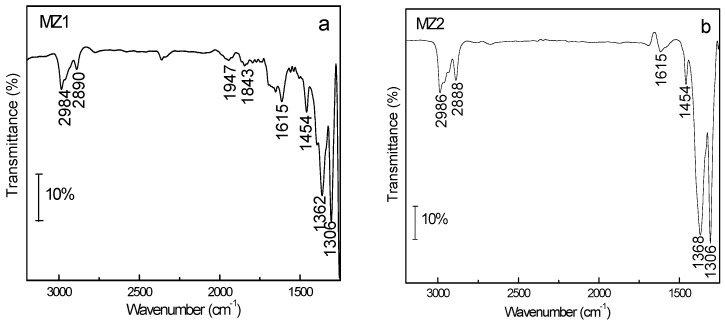
*In-situ* IR result of NPYR adsorbed on (**a**) MZ1 and (**b**) MZ2 samples.

The evidence of MZ1 and MZ2 sample adsorbing NPYR is further provided by the *in-situ* FTIR experiment as demonstrated in Figure 5. In the spectrum of the MZ1 sample, some special IR signals of NPYR appear. Among them, the 2984 cm^−1^ and 2890 cm^−1^ bands are the ν_as_ (CH_2_) and ν_s_ (CH_2_) vibrations in the pyrrolidinyl structure of NPYR, and the 1615, 1454 cm^−1^ bands can be regarded as the ν_as_ (N=O) and ν_3_(NO_2_) vibrations in the MZ1 sample. The bands at 1362 cm^−1^ and 1306 cm^−1^ are considered as signals of nitrite and the vibration of the C-N bond. The structure of SBA-15 is unsuitable to adsorb volatile nitrosamines, due to the lack of fine geometric confinement [22,23,24]. Introducing a zeolite fragment in the composite covers this deficiency; apart from the necessary micropores, tervalent aluminum cation in the adsorbent may possess an attraction function toward the N-NO group of volatile nitrosamines to enhance the adsorption, because the N-NO functional group of nitrosamines possesses a negative charge [35,36,37]. The *in-situ* IR spectrum still showed the adsorption of NPYR on the MZ2 sample, and the FTIR peaks were similar to that of the MZ1 sample (Figure 5), indicating a similar adsorptive mode of NPYR on MZ2 to that on MZ1 sample.

### 2.3. Application of Hierarchical Composites as the Filter Additive

Table 3 provides the normal smoking data of the cigarettes containing porous additive in the filter. Adding 40 mg of activated carbon into the cigarette filter brought a slight decrease to the tar (NFDPM), nicotine, TPM and CO in the mainstream smoke, while the level of water remained constant. MZ1 additive in the filter did not change the average puff number and CO content, but lowered by about 10% the tar, nicotine and TPM. The MZ2 sample showed a better performance in reducing the tar (17%), nicotine (16.8%) and TPM (16.9%) though it had a relatively narrow pore size.

**Scheme 1:**
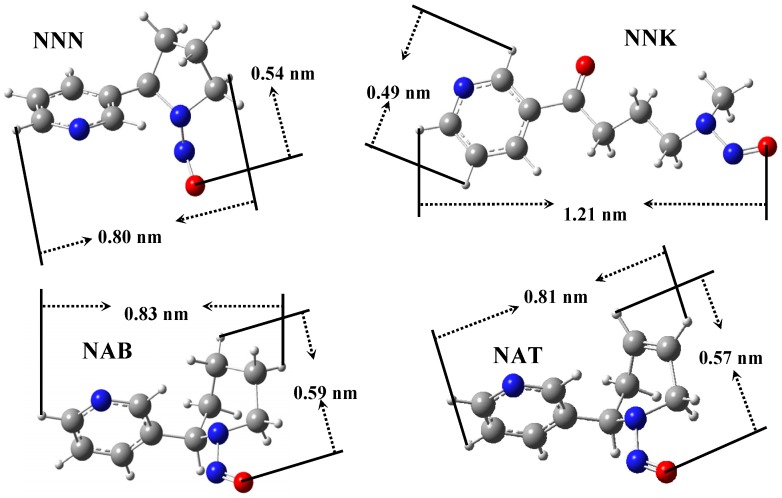
The molecular structure of four TSNA components.

**Scheme 2:**
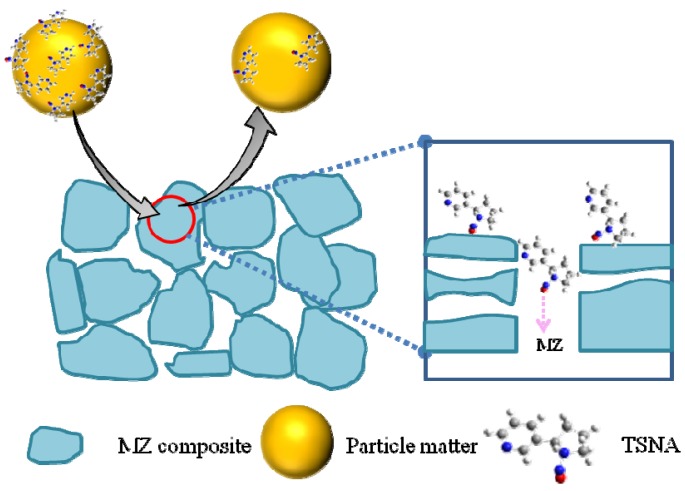
The mechanism of removing TSNA from smoke particles by porous adsorbent.

Table 4 reveals the performance of porous additives in the cigarette filter in reducing the TSNA level of mainstream smoke. Activated carbon failed to decrease the amounts of TSNA in smoke, *i.e.*, the removal of NAB and NNK were only 4.1% and 1.8% while no NAT or NNN was trapped. In contrast, the MZ1 sample trapped NNN of 15.3% and NAB of 13.2%, but its effect on NAT and NNK was not obvious. This result was similar to the report of HZSM-5 zeolite put into the tobacco rod of the cigarette (18%, [17,18]). An obvious function of trapping TSNA was observed on the MZ2 sample though it had a smaller average pore size than MZ1; it had an effective and uniform function for reduction of four TSNA components, reducing about 28% of NAT and NNK, 30.7% of NNN and 31.1% of NAB, respectively (Table 4), which was close to that for zeolite NaY in the tobacco rod of cigarette (31.3%, [17]). TSNA has a larger molecular diameter than volatile nitrosamines, for instance NNN has a molecular size equaling 0.54 × 0.80 nm^2^. Moreover, most of the TSNA in smoke adhered on the particulate phase whose smoke particles have diameters of μm grade [3]. Consequently, neither zeolite nor mesoporous silica can directly adsorb the TSNA in smoke because their microporous or mesoporous channels only have a pore size of nm grade. Unlike the simple adsorption of volatile nitrosamines in the gaseous phase, where the carrier gas passes through, while the target carcinogen is trapped by the porous adsorbent, capturing TSNA in cigarette smoke by the filter additive involves several steps. The first, the adsorbent should be able to intercept the particle [15,38,39,40], and then contact with the target so that the TSNA adhered on the surface of the smoke particle can be captured by the adsorbent. After the adsorption, the purified smoke particle should leave in order to empty the adsorptive site for the successive adsorption procedure [41]. In other words, this several-step adsorption-purification resembles that of “washing a car”. To realize this function, the adsorbent should have the necessary hierarchical structure, not only the basic micropore or mesopores to adsorb TSNA, but also the optimal textual property to intercept and flick the particle. That is, the adsorbent should have an elastic collision with the smoke particle and capture TSNA from the particle during the collision; for which the morphology of adsorbent becomes the key factor, and a suitable particle size enables the elastic collision to be realized [16]. The special morphology of the MZ2 sample may promote the interception of the particles in the smoke. It appears that a lot of MZ2 particles deposit together (Figure 2c), and their particle size is in the range of 0.5–2 μm, which may match the sizes of the particulate phase in smoke (from 0.1 to 1.0 μm) so that the elastic collision easily happens. Besides, the NaY zeolite fragments in the MZ2 composite kept a pore size of 0.8 nm, considerably larger than that in the MZ1 sample (0.5 nm) so that the TSNA molecule easily inserts its N-NO group inside the channel to be adsorbed [28]. The four components of TSNA, NAB, NAT, NNK, and NNN have different molecular structures (Scheme 1) but their reduction by the MZ2 additive was similar (28%–31%, Table 4) within the experimental error. This phenomenon implies the existence of an “insertion model” in the adsorption of TSNA by the hierarchical additive, in which the TSNA molecule inserts its N-NO group inside the channel of the adsorbent because of the electrostatic inducement from the cations in the channel [42]. In order to fully assess the function of porous additives in cigarette filters in the selective reduction of TSNA, the ratio of TSNA to tar (NFDPM) was calculated from the data of Table 3 and Table 4. The value of the control cigarette is 5.2, and whereas it is 6.0 or 5.4 for activated carbon or MZ1 so these two additives cannot trap the TSNA adhered on the particle phase of the smoke. A small value of 4.4 is found for the MZ2 additive, mirroring the selective reduction of TSNA by this hierarchical composite. A suitable state of aggregation and distribution of additive in the filter or tobacco rod is important for its function in capturing TSNA that adheres on the particles. The particles in the smoke have a size of 0.1–1 µm [4], and they can easily pass through the accumulated pores of MZ2 among the aggregations after collision with the additive particles. Through this elastic collision model, the MZ2 additive can effectively capture the TSNA adhered on the smoke particle but avoids the cover of these particles and deactivation (Scheme 2). Here the special aggregation and distribution state of MZ2 plays a crucial role. The particles in the aggregation have a size less than 2 µm so that they can collide elastically with the smoke particle, meanwhile their micropores and mesopores can trap the TSNA through electrostatic interaction [18,20,28], and the adsorption will be complete once the smoke particle is flicked [41]. Only the TSNA adhering on the external surface of the smoke particle is trapped while that inside the particle cannot be removed such that the removal of TSNA in the cigarette smoke by the filter additive is inevitably limited [5,17,41], and the reduced proportion depends on the initial level of TSNA in the smoke.

**Table 3:** Smoking data of cigarette X148 and the one with additives in the filter.

Sample	NFDPM (mg cig^−^^1^)	Nicotine (mg cig^−^^1^)	TPM (mg cig^−^^1^)	Water (mg cig^−^^1^)	Puff No.	CO (mg cig^−^^1^)
Control	10.6	0.95	13.0	1.44	7.3	9.9
Carbon	9.6	0.92	12.0	1.45	7.3	9.6
MZ1	9.4	0.89	11.9	1.56	7.3	9.9
MZ2	8.8	0.79	10.8	1.24	7.1	9.9

Notes: NFTPM: nicotine-free dry particulate matter; TPM: total particulate matter.

**Table 4:** The results of the porous materials as the filter additives to reduce the TSNAs in the mainstream smoke.

Sample	NAB		NAT		NNK		NNN	
	Amount (ng cig^−1^)	Removed (%)	Amount (ng cig^−1^)	Removed (%)	Amount (ng cig^−1^)	Removed (%)	Amount (ng cig^−1^)	Removed (%)
Control	3.18	-	26.8	-	11.1	-	14.4	-
Carbon	3.05	4.1	28.3	-	10.9	1.8	15.5	-
MZ1	2.76	13.2	24.4	9	11.6	-	12.2	15.3
MZ2	2.19	31.1	19.3	28	7.97	28.2	9.98	30.7

Notes: TSNAs = tobacco specific nitrosamines; NAB = *N*′-nitrosoanabasine; NAT = *N*′-nitrosoanatabine; NNK = 4-(methylnitrosamino)-1-(3-pyridyl)-1-butanone; NNN = *N*′-nitrosonornicotine.

**Figure 6:**
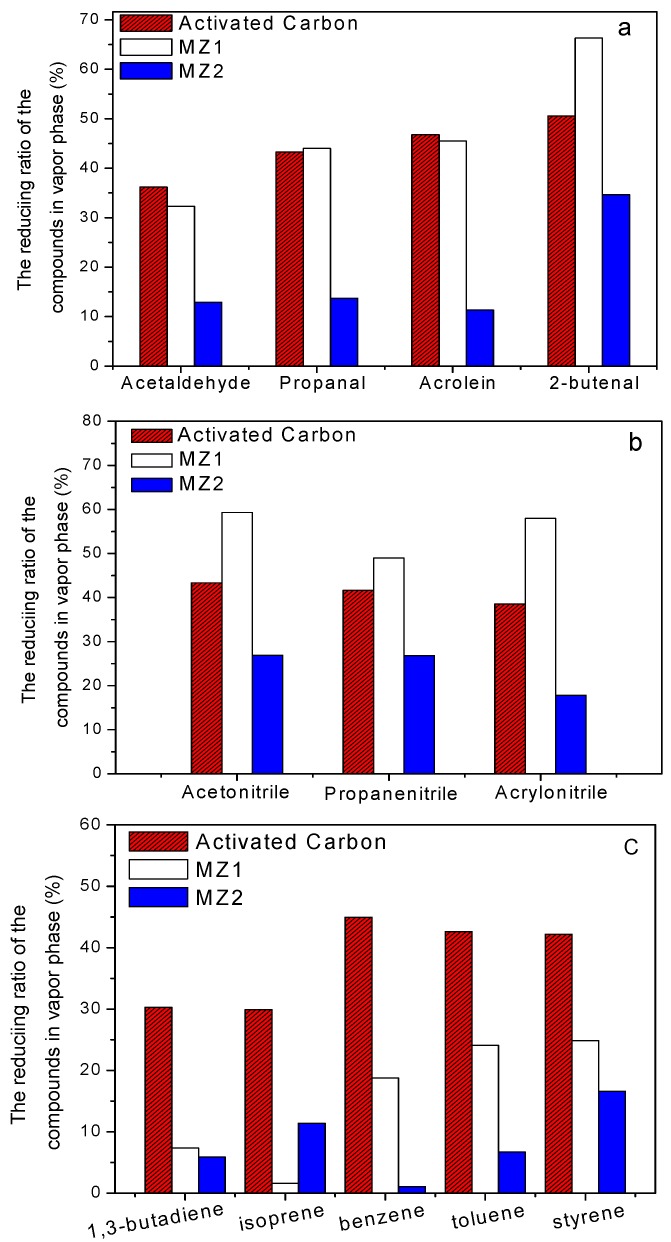
The effects of porous materials in trapping vapor phase compounds as filter additives. (**a**) Acetaldehyde, propanal, acrolein, and 2-butenal; (**b**) Acetonitrile, propanenitrile, and acrylonitrile; (**c**) 1, 3-butadiene, isoprene, benzene, toluene, and styrene.

Adsorption of vapor phase compounds in the mainstream smoke by porous filter additives is displayed in Figure 6. These substances have a molecular size smaller than the pore of the three additives used here. Activated carbon is very effective in trapping vapor substances [2], dramatically better than the MZ2 composite. The MZ1 sample only had a higher ability than activated carbon in eliminating 2-butenal (66.3%) and nitrile compounds, implying its special affinity to the functional group of -C≡CH. The performance of MZ1 in adsorbing propanal and acrilein was similar to that of activated carbon, but inferior to carbon in capturing other components such as toluene, styrene, benzene, and 1, 3-butadiene (Figure 6c). It seems that the pore structure and morphology of MZ1 and MZ2 do not have any function in this situation due to the complex composition of cigarette smoke, which will spur further design of new materials.

## 3. Experimental Section 

### 3.1. Reagents and Sample Synthesis

*N*-nitrosodimethylamine (NDMA), *N*-nitrosopyrrolidine (NPYR) and *N*-nitrosohexamethyleneimine (NHMI) were purchased from Sigma and dissolved in dichloromethane in a volume ratio of 1:19 [25]. Zeolite NaY (Si/Al = 2.86) and HZSM-5 (Si/Al = 12.5) were commercially available powders, the activated carbon with average pore size of 3–5 nm was made from coconut shell [2,43], and all reagents used were of AR grade. The cigarette used in this investigation was a Virginia type purchased from the market.

The hierarchical composite named MZ1 was prepared using tetraethyl orthosilicate (TEOS) and HZSM-5 (Si/Al = 12.5) as silica and zeolite precursors, respectively. About 2.0 g Pluronic P123 (ethylene oxide (EO)/propylene oxide (PO) triblock copolymer, EO_20_PO_70_EO_20_, Aldrich, Shanghai, China) was mixed with 60 g 2M HCl, 15 g H_2_O in a beaker and stirred at room temperature. When the solution turned clear, 1.2 g HZSM-5 was added to the solution, followed by stirring of 3 h at 313 K. After adding 4.25 g TEOS into the solution and stirring another 24 h at 313 K, the synthesized gels were autoclaved at 373 K for 24 h. The as-synthesized sample was filtered, washed with water and air-dried, followed by t calcination at 823 K in a flow of air for 6 h to remove the template.

To prepare the sample of MZ2, about 3 g SiO_2_ (100 mesh) was dissolved in 45 mL 0.56 M NaOH solution as the silicic precursor at 333 K. After that, 3 g NaY zeolite was added to this solution and stirred for 2 h. This solution was labeled as solution A. Solution B was the aqueous solution of CTAB that contained about 4.55 g CTAB in 25 g H_2_O. Then, solution B was dropped into solution A slowly with continuous stirring. After dropping the solution, the pH value of this mixture was adjusted to 11 by using 2 M HCl. The solution was stirred for another 6 h, and removed to an autoclave at 373 K for 72 h. The as-synthesized material was filtered, washed and air-dried, then calcined in a flow of air for 5 h at 823 K.

### 3.2. Characterization

The samples were characterized by powder X-ray diffraction recorded on an ARL XTRA diffractometer with Cu Kα radiation in the 2-theta ranges from 0.5 to 8 degrees or from 5 to 70 degrees. To obtain the FTIR spectrum of the sample, a compressed KBr pellet containing 3 wt% of sample was used and the spectrum was recorded on a Bruker 22 infrared spectrophotometer in 4000–400 cm^−1^ with a resolution of 4 cm^−1^. The nitrogen adsorption-desorption isotherms at 77 K were measured in a Micromeritics ASAP 2020 system, and about 100 mg of sample (20–40 mesh) was evacuated at 573 K for 4 h prior to the test. The Brunauer-Emmett-Teller (BET) specific surface area (S_BET_) was calculated using adsorption data acquired at the relative pressure (*P*/*P*_0_) range of 0.05–0.22 and the total pore volume determined from the amount adsorbed at a relative pressure of about 0.99. The pore size distribution (PSD) curve was calculated from the analysis of the desorption branch of the isotherm using the Barrett-Joyner-Halenda (BJH) algorithm [22,24].

Adsorption of volatile nitrosamine like NPYR was performed in a stainless steel micro-reactor [28]. A 5 mg sample (20–40 mesh) was filled in one end of the reactor and sealed by glass wool to fix the position. Then, this part was inserted deeply into the injector port of Varian 3380 GC while another end connected with the separation column in the GC [28]. The sample was directly heated to 338 K in a helium flow at a rate of 30 mL min^−1^, and the NPYR solution was pulse injected with amounts of 2 μL each time. The TCD of GC analyzed the gaseous effluent, and the decrement in the ratio of solute to solvent utilized to calculate the adsorbed amount [14,28].

*In situ* FTIR experiments were performed in a home-built IR cell with CaF_2_ windows [38]. A Bruker 22 spectrometer, with a resolution of 4 cm^−1^, was used. The samples disc, with a density of 15 mg cm^−2^, was activated at 773 K in N_2_ flow for 2 h, and then the background spectrum was collected on the activated adsorbate-free sample. After that, 0.4 μL NPYR was introduced into the IR cell at 493 K and then the sample was purged with a nitrogen flow for 10 min to remove the physically adsorbed nitrosamines prior to recording the FTIR spectrum.

In order to assess the performance of porous composite as filter additive, a 40 mg sample (2–40 mesh) was added into the middle of the cigarette filter [39]. All of the cigarettes were conditioned for 48 h at 293 K and 60% R.H. before testing. Three cigarettes were smoked using a 20-port smoking machine (Borgwaldt RM20; Borgwaldt KC GmbH, Hamburg, Germany.) with puffing parameters of: 35 mL puffs of 2 s duration every 60 s. All cigarette samples were smoked in triplicate. The particulate phase of the smoke was separated from the gas phase by passing smoke through a standard Cambridge filter pad. After the last replicate was smoked, the Cambridge pad filter was transferred from the pad holder to a 50-mL centrifuge tube. The filter holder was wiped twice with a quarter of a filter and added to the centrifuge tube as well. Finally, 30 mL of methanol and 100 μL of an internal standard solution were added [15,40]. The centrifuge tube was shaken for 30 min at 200 rpm on an orbital shaker. No further clean-up was carried out. About 1 mL of liquid from the centrifuge tube was transferred to an autosampler vial and analyzed by LC-MS/MS.

Three cigarettes were smoked by using a single-port Burgwaldt smoking machine under ISO standard conditions. The mainstream smoke was collected by a Tedlar bag through a Teflon tube (SKC INC., Covington, GA, USA). A set of HP 6890 GC and HP 5972 MSD was utilized to analyze the compounds in the vapor phase. Peak assignments in the GC-MS chromatograms were made using an on-line library attached to the equipment workstation, the Wiley Registry of Mass Spectral Data, 6th Edition, by F.W. McLafferty.

## 4. Conclusions

Two hierarchical composites named MZ1 and MZ2 were synthesized successfully. Zeolite HZSM-5 or NaY fragments can survive from a synthetic solution of SBA-15 or MCM-41, producing less influence on the mesoporous structures of the resulting hierarchical composite. The specific filter additives, MZ1 and MZ2 exhibit a better ability than activated carbon in reducing some carcinogenic compounds like TSNA in mainstream smoke. Activated carbon has little effect on reducing the amount of TSNA, but MZ2 can trap 29.5% of the carcinogens from mainstream smoke. 
